# Case Report: Marburg variant of multiple sclerosis and review of its complicated treatment

**DOI:** 10.3389/fimmu.2026.1816028

**Published:** 2026-05-14

**Authors:** Anjan Bhattarai, Ahmad Awwad, Anwar Zahran, Mustafa Abdul Kareem, Aakriti Subedi, Anna V. Bite

**Affiliations:** 1 Department of Neurology, St. Vincent Mercy Medical Center, Toledo, OH, United States; 2 Department of Medicine, An-Najah National University, Nablus, Palestine; 3 Department of Nursing, Owens Community College, Toledo, OH, United States

**Keywords:** biopsy, high dose cyclophosphamide (HiCy), immunotherapies, Marburg variant of MS, outcomes

## Abstract

Marburg variant of multiple sclerosis (MS) is a rare, fulminant demyelinating disorder characterized by rapid neurological decline and notable lack of established standardized treatment guidelines. We describe a 46-year-old woman presenting with progressive cognitive and behavioral changes and multifocal CNS lesions initially refractory to corticosteroids, plasmapheresis (PLEX), and intravenous immunoglobulin (IVIg). Brain biopsy confirmed aggressive MS-spectrum demyelination. Escalation to high-dose cyclophosphamide resulted in radiologic stabilization but was limited by severe, refractory cytopenia. Given persistent disease activity and intolerance to infusion-based therapy, transition from ocrelizumab to subcutaneous ofatumumab was planned as a pragmatic maintenance strategy; however, treatment initiation was delayed. The patient subsequently experienced rapid clinical deterioration marked by recurrent aspiration events and functional decline, ultimately leading to death following transition to comfort-focused care. This case highlights the therapeutic challenges of Marburg MS, including treatment-limiting toxicity, logistical barriers to anti-CD20 therapy, and the critical importance of timely, individualized immunosuppressive strategies.

## Introduction

The Marburg variant of MS, first described by Otto Marburg in 1906, is a rare, fulminant extreme of the MS spectrum. Clinically, it is characterized by rapid neurological deterioration over days to weeks. Radiologically, it typically shows extensive, often confluent lesions in the cerebrum, brainstem, or spinal cord that may enhance, be surrounded by edema, or both, which mimics acute disseminated encephalomyelitis (ADEM), tumefactive demyelination, or even high-grade glioma or lymphoma in the early stages (1–3). Historically, mortality was high when treatment options were limited to corticosteroids and supportive care (4). Despite advances in immunotherapy, outcomes remain highly variable, and evidence-based treatment guidelines are lacking. We hereby present a treatment-challenging case of Marburg variant of MS and a review of treatment options reported in the literature.

## Case description

**Figure 1:**
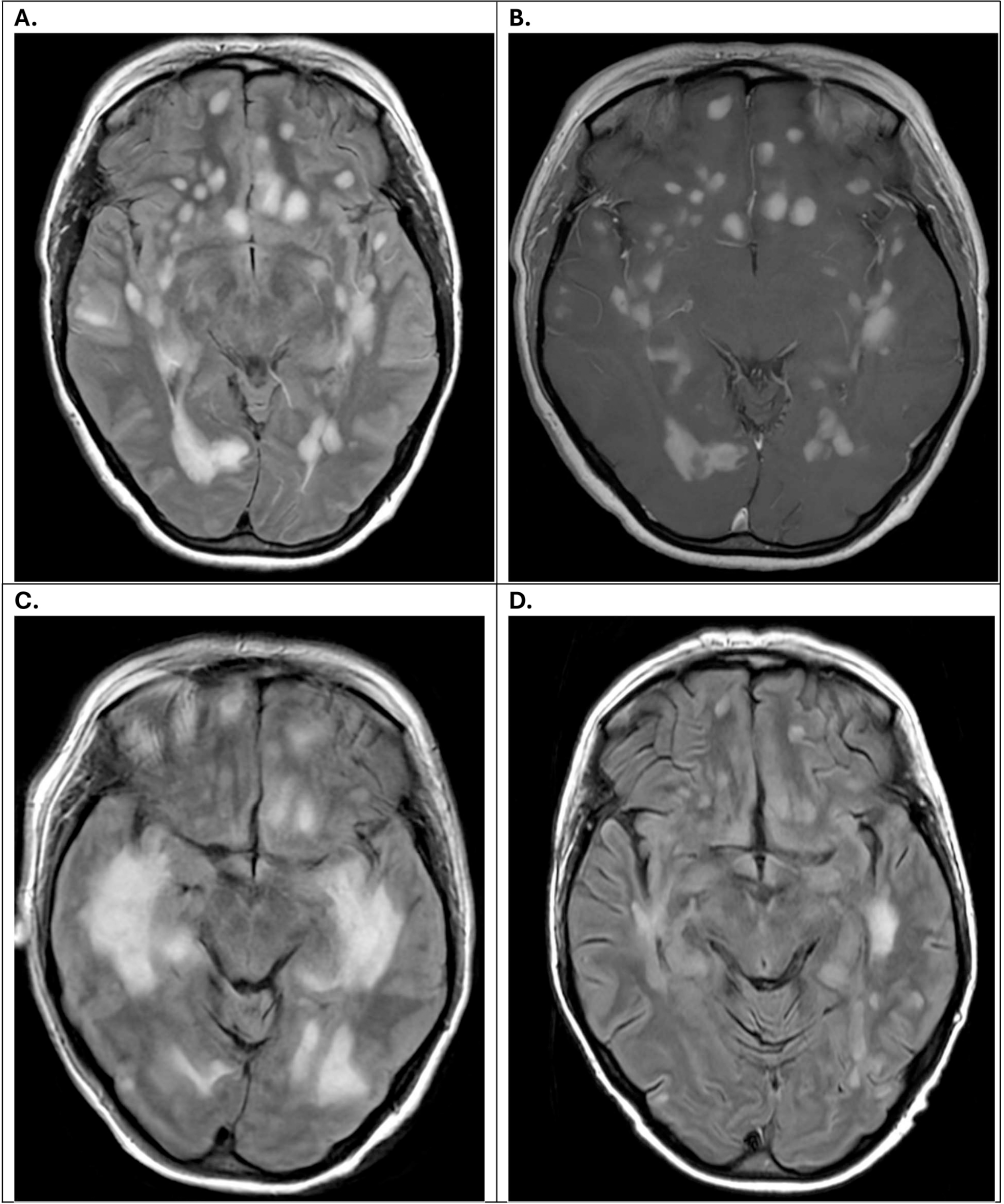
MRI brain images at initial presentation **(A, B)**, post 5 days of high dose steroids, plasmapheresis X 5 sessions and IVIg X 5 days **(C)**, post HiCy X 2 doses **(D)**. **(A)** Initial MRI brain axial T2 FLAIR image showing extensive supratentorial lesions. **(B)** Initial MRI brain axial T1 post contrast image showing extensive demyelination along the supratentorial lesions. **(C)** MRI brain axial T2 FLAIR image post high dose IV steroids for 5 days, plasmapheresis X 5 sessions, and IVIg X 5 sessions showing progression of the lesions with increased size of the supratentorial lesions. **(D)** MRI brain axial T2 FLAIR section post HiCy X 2 doses showing stabilization of the supratentorial lesions with decrement of the size.

A 46-year-old female with a history of anxiety and depression presented with a gradual cognitive decline of 3 weeks’ duration followed by agitation and confusion. Her initial MRI showed scattered T2 lesions in white matter, including periventricular lesions. Repeat MRI Brain after a few days showed new lesions involving both supratentorial and infratentorial lesions, along with enhancing lesions. MRI Cervical and Thoracic spine showed several non-enhancing lesions in the spinal cord (**Figures 1A, B**). Extensive serum and CSF workup was unremarkable except for the CSF, which showed an inflammatory picture with mildly elevated protein, WBC, and unpaired oligoclonal bands (**Table 1**). She was treated with 5 days of high-dose steroids, plasmapheresis X 5 sessions, and IVIg X 5 days without clear clinical and radiological improvement as lesions continued to grow (**Figure 1C**).

**Table 1:** Summary of serum and cerebrospinal fluid workup.

Lab workup(serum)	Results
WBC	1 x 10^3^ at presentation; 0.1 X 10^3^ for a week after 2nd dose of cyclophosphamide despite receiving G-CSF (filgrastim) on alternate days for 4 doses.
Antinuclear antibody (ANA) Rheumatoid arthritis (RA factor)	Negative
Treponema pallidum antibody	Non-reactive
Human immunodeficiency virus immunoassay (HIV)	Non- reactive
Erythrocyte sedimentation rate (ESR)	13
C reactive protein (CRP)	7.3
Neuromyelitis optica/Aquaporin 4 antibody (NMO/AQ4 Ab)	Negative
Myelin oligodendrocyte antibody (MOG Ab)	Negative
Thyroid peroxidase antibody (TPO)/Anti thyroglobulin antibody	Negative
QuantiFERON gold	Negative
Angiotensin-converting enzyme (ACE)	Normal
Lab workup (CSF)	
WBC (lymphocytes 95%)	43
RBC	1
Protein	69.4
Oligoclonal bands	Positive, 3 bands
IgG index	0.5
Meningitis encephalitis panel	Negative
Herpes and Varicella	Negative
VDRL	Non-reactive
Cytology	Negative for malignancy
Cryptococcal antigen	Negative
Borrelia burgdorferi (Lyme) antibody	Negative
Toxoplasma gondii antibody	Negative
West Nile virus antibody	Negative
Angiotensin-converting enzyme (ACE)	Negative
Neuromyelitis optica/Aquaporin 4 antibody (NMO/AQ4 Ab)	Negative
Myelin oligodendrocyte antibody (MOG Ab)	Negative
Mayo-clinic autoimmune and demyelinating panel (ENC2)	Negative

As the patient did not improve with treatment, brain biopsy was performed. Histopathological examination of the brain biopsy revealed diffuse, macrophage-rich white matter demyelination with abundant reactive astrocytes and few lymphocytes. Immunohistochemical analysis demonstrated a predominantly T-cell infiltrate, with CD3-positive lymphocytes distributed both in perivascular and parenchymal compartments. CD20-positive B cells were present but represented a distinctly minor component of the inflammatory population. Glial fibrillary acidic protein (GFAP) highlighted diffuse reactive gliosis throughout the specimen. Neurofilament staining showed appropriate axonal preservation. Luxol fast blue/periodic acid–Schiff (LFB/PAS) staining revealed extensive myelin loss within the white matter with only sparse residual myelinated fibers. Simian virus 40 (SV40) immunostaining was negative, excluding JC virus–associated progressive multifocal leukoencephalopathy. Taken together, the morphological and immunophenotypic profile was consistent with an active demyelinating plaque favoring an aggressive form of multiple sclerosis.

Ultimately, she was started on high-dose cyclophosphamide and received two doses before it was stopped due to significant cytopenia not responding to filgrastim (G-CSF). Repeat MRI of the brain post 2 doses of high-dose cyclophosphamide (HiCy) showed decreased lesion size with no new or enhancing lesions; however, treatment was discontinued due to refractory cytopenia (**Figure 1D**). At the time of discharge, the patient continued to remain confused and struggled with recall and short-term memory.

Over subsequent follow-up visits, her recovery was extremely slow. Her husband reported that she recognized her children and followed simple one-step instructions, but remained agitated when exposed to unfamiliar environments or people. Plans were initially made to start her on Ocrelizumab infusions in an outpatient clinic but given her tendency to become agitated outside her home environment, the team pivoted to considering Ofatumumab as a subcutaneous injection that could be administered at home. Tragically, before that medication could receive approval, she experienced a sudden and rapid decline marked by frequent falls and aspiration events. Her family opted for comfort-focused care, and she passed away within the following weeks.

## Discussion and review of treatment

Our patient illustrates how heterogeneous and misleading the clinical picture can be. Rather than a classic relapsing-remitting course with focal deficits, she presented with a subacute cognitive and behavioral syndrome, followed by rapid neurological decline and multifocal CNS involvement on MRI. The evolution of her imaging findings, together with inflammatory CSF and a negative infectious, autoimmune, and paraneoplastic workup, progressively shifted suspicion toward an aggressive demyelinating process. In this context, brain biopsy, although invasive, was ultimately decisive, confirming an MS-spectrum demyelinating pathology and justifying escalation to aggressive immunosuppression (5, 6). This sequence highlights a key point repeatedly emphasized in prior Marburg MS reports: early recognition and early commitment to high-intensity immunotherapy are crucial (1–3).

The current treatment landscape for Marburg MS is based almost entirely on case reports/series (**Table 2**), and retrospective reviews; no randomized trials or formal guidelines exist. Nonetheless, when these observations are read with a quasi–meta-analytic mindset, some patterns emerge. First-line therapy in nearly all reported cases consists of high-dose intravenous methylprednisolone followed by an oral taper. In steroid-refractory cases, plasmapheresis and, less consistently, intravenous immunoglobulin (IVIg) were typically added (4, 7, 8). While this stepwise approach is appropriate and standard, almost all published Marburg MS cases did not stabilize with this “conventional” escalation alone; new lesions often continued to appear, and disability progressed similarly to the course observed in our patient (8, 9).

**Table 2:** Comparative summary of treatment and outcomes of Marburg cases reported in the literature.

Cases/references	Population/disease pattern (clinical/radiological)	Induction beyond steroids ± PLEX/IVIg	Maintenance/long-term strategy	Outcome & key message
Present case	Middle-aged woman (46 years);cognitive/behavioral issues at onset; multiple supratentorial, infratentorial, and spinal cord lesions.	No response to IV steroids, PLEX, IVIg; HiCy started but stopped early due to severe refractory cytopenia.	Ofatumumab was planned as long-term DMT but could not be started due to untimely demise of the patient.	Slow, partial recovery with residual disability at discharge; illustrates effect of incomplete induction and delayed B-cell depletion in real-world practice. Lack of early initiation of antiCD20 therapy which could have possibly prevented further clinical decline if it was started on time.
Nozaki et al., 2010 (7)	26-year-old woman; initial symptoms: subtle left-hand weakness and decreased sensation below T4 dermatome, later progression to near quadriplegia; multiple brainstem and subcortical lesions.	HiCy 50 mg/kg/day X 4 days with G-CSF support after failed steroids, IVIg and PLEX.	No specific maintenance DMT reported.	Marked MRI and significant clinical improvement at 5 months with minimal residual right-sided weakness; able to ambulate independently. Prototypical HiCy “success story” contrasted with truncated HiCy in our case.
Koska et al., 2021 (9)	26-year-old patient; initial presentation with bilateral optic neuritis (bilateral blurred vision, reduced color discrimination, pain with eye movement; VA left: 20/80, right: 20/40); >100 gadolinium-enhancing lesions (supratentorial, infratentorial and spinal cord).	HiCy 50 mg/kg/day X 4 days after failure of IV steroids and PLEX.	Early ocrelizumab as maintenance.	Near-complete clinical and radiologic remission; clinically stable without relapses at 6 months; only residual symptom was slightly impaired visual acuity (left: 20/30, right: 20/30); no gadolinium enhancement detectable at 44 days post-cyclophosphamide. “ideal” HiCy → early anti-CD20 induction–maintenance model that our case aimed for but could not be achieved.
Alshamrani et al., 2025 (10)	Subacute onset of right leg weakness and numbness, with later rapid progression to encephalopathy, aphasia, dysphagia, and bilateral weakness; multiple round/oval T2/FLAIR hyperintense lesions in pons, periventricular and subcortical white matter, left optic nerve involvement with new lesion development and enlargement on follow-up.	HiCy 600 mg/m²/day IV. (alternate day, 5 doses) after failure of IV steroids, PLEX and IVIg.	Rituximab	Improvement in expressive aphasia and ability to follow two-step commands 5 days after discharge; EDSS improved from 4 at discharge to 2 at 2-month follow-up.
Manuel et al., 2021 (11)	55-year-old woman; Marburg MS with rapid deterioration, initial presentation with new onset seizure, AMS with low GCS; extensive brainstem/left frontal lesions.	single dose mitoxantrone 12 mg when steroids and IVIg failed.	Short azathioprine trial (stopped due to cytopenia); no clearly defined long-term high-efficacy DMT.	Radiologic regression at 3 weeks and good functional recovery; no relapse at 2 years — supports early mitoxantrone rescue in fulminant disease.
Capet et al., 2022 (12)	31-year-old woman; initial presentation with dysarthria and left facial palsy progressing to left proportional hemiplegia, left babinski sign, complete left anesthesia, anosognosia with left-sided visuospatial hemineglect, and fluctuating altered consciousness within 24 hours; large right frontal pseudonodular lesion.	Very early mitoxantrone (12 mg/m²; 20 mg/month for 2 doses) following no response to IV steroids and PLEX.	Anti-CD20 drug as maintenance (individual drug name not mentioned).	Complete remission with durable stability; five-fold decrease in frontal lesion size at 3 months; complete clinical remission (EDSS: 1) at 6 months without cognitive impairment — illustrates mitoxantrone-based induction followed by B-cell depletion as an alternative to HiCy.
Gobbin et al.,2017 (21)	51-year-old-woman; initial presentation with rapidly worsening left hemiplegia, vision loss, and cognitive impairment; multiple large bilateral periventricular, right cerebellar peduncle and pons lesion.	High dose IV steroids and PLEX with no response.	Alemtuzumab (12mg/day for 5 days)	Patient became awake and responded to external stimuli and showed initial motor recruitment of right arm in the following weeks post alemtuzumab infusion. FU after 2 months: Repeat MRI brain: stable lesion load with no enhancement. Further clinical improvement of upper limb motor function, cognition, and vision.

Abbreviations: PLEX, plasmapheresis; IVIG, intravenous immunoglobulin; HiCy, high-dose cyclophosphamide; IVMP, intravenous methylprednisolone; DMT, disease-modifying therapy; MS, multiple sclerosis; GCS, Glasgow Coma Scale; AMS, altered mental status; G-CSF, granulocyte colony-stimulating factor; EDSS, Expanded Disability Status Scale; VA, visual acuity.

For this reason, many authors advocate an early transition to a second tier of truly aggressive immune therapies (high-dose cyclophosphamide (HiCy), mitoxantrone, anti-CD20, alemtuzumab, autologous hematopoietic stem cell transplant) once a fulminant MS variant is suspected (7–16).

Multiple reports describe patients with extensive, gadolinium-enhancing lesions and severe clinical deficits who experienced dramatic radiologic and functional improvement after high dose cyclophosphamide (HiCy) regimen, typically 50 mg/kg/day for four consecutive days (7, 9, 10). In some cases, long-term follow-up suggests sustained remission when HiCy is followed by an appropriate maintenance strategy (8). Cyclophosphamide is a cell cycle nonspecific cytotoxic agent that exerts its effects on both B cells and T cells, suppressing both humoral and cell-mediated immunity, with major risks including malignancy (bladder cancer), hemorrhagic cystitis, severe cytopenia, and gonadotoxicity (17, 18).Despite the known downside of cyclophosphamide, attestations to its efficacy, low cost, extensive availability, and experience with it keep it among the first-choice agents for treating aggressive Marburg variant of MS; thus, it was chosen for our patient.

Mitoxantrone is another cytotoxic agent with reported successes in malignant or Marburg-type MS. It inhibits the proliferation of B cells, T cells and suppresses TH1-mediated cytokines (e.g., tumor necrosis factor [TNF]-α, interleukin [IL]-12) (19, 20).The most commonly used regimen is the induction protocol (three doses of 12 mg/m^2^ monthly followed by six monthly infusions of the same dose until a maximum of 110 mg/m^2^ to 120 mg/m^2^ has been reached). Several case reports and small series describe patients who achieved long-term stability after early mitoxantrone, either alone or in combination with other immunosuppressants (11, 12). However, its well-recognized cardiotoxicity, cumulative dose limits, gonadal dysfunction in young patients and risk of idiosyncratic leukemia make it a challenging choice, particularly in middle-aged patients and in healthcare systems where rigorous long-term monitoring is difficult to guarantee (22). Conceptually, mitoxantrone remains a reasonable alternative when cyclophosphamide is contraindicated or not tolerated, but its risk–benefit profile must be carefully individualized.

B-cell–depleting (anti-CD20) therapies have transformed the treatment of relapsing–remitting and highly active MS (9, 23, 24). Although data in Marburg MS are limited, their use is biologically plausible given the central role of B cells in MS pathogenesis. They have been used as acute rescue therapy or maintenance following induction with cyclophosphamide, mitoxantrone, or autologous hematopoietic stem cell transplantation (8–10, 12). Early initiation appears critical to prevent irreversible tissue injury. In our patient, subcutaneous ofatumumab was considered as a maintenance strategy due to ongoing agitation, intolerance of infusion-based treatments, and the need for a practical long-term approach. The other important rationale behind switching to ofatumumab was that following subcutaneous administration, ofatumumab achieves rapid and profound B-cell depletion, with median time to B-cell depletion (CD19+ count <10 cells/μL) of approximately 14 days after the first dose (25).

This aligns with an emerging induction–maintenance paradigm, although in this case the induction phase was necessarily incomplete (9, 10, 12).Such scenarios, in which optimal protocols are constrained by toxicity and feasibility, are underrepresented in the literature yet highly relevant to routine clinical practice. Sadly, ofatumumab was never initiated. Before approval could be obtained and treatment started, the patient experienced sudden and severe clinical deterioration, and her family made the decision to transition to comfort care.

Alemtuzumab and Autologous Hematopoietic Stem Cell Transplant (AHSCT) represent an even more aggressive tier of immune reconstitution therapies. Alemtuzumab is a humanized monoclonal antibody directed against CD52, a surface antigen expressed at high levels on T and B lymphocytes, that rapidly depletes lymphocytes, producing sustained depression for up to 1 year (13, 26).A course of treatment with alemtuzumab consists of 5 consecutive days in the first year and 3 in the second year. Alemtuzumab has been used in a small number of fulminant or tumefactive MS cases with encouraging outcomes, but carries significant risks of secondary autoimmunity (e.g., thyroid disease, idiopathic thrombocytopenic purpura, or Goodpasture syndrome) and infection (27).

AHSCT is now recognized as a highly effective option for selected patients with aggressive MS, including malignant variants, with registry data demonstrating substantial reductions in inflammatory activity and disability progression in appropriately chosen candidates (15, 16, 28). The rationale for this severe approach rests on the ability of this treatment to produce a complete immune system reset, effectively eradicating the disease-causing immune cells using intense immunosuppression and reestablishing a new immune system derived from autologous stem cells (14). However, it requires specialized centers, intensive supportive care, and meticulous patient selection. For many patients with Marburg MS worldwide, these options remain aspirational rather than realistically available.

## Recommendations/conclusions

There is a desperate need for collaborative registries and pooled analyses of Marburg MS cases. Randomized trials are unlikely, but systematically collected data could help clarify which patients benefit most from HiCy, mitoxantrone, B-cell depletion, AHSCT, or combinations of these treatments. This would help the field progressively transition from anecdotal to more structured, evidence-based care, thereby improving patients’ recovery and clinical outcomes.

## Data Availability

The datasets presented in this article are not readily available because of ethical and privacy restrictions. Requests to access the datasets should be directed to the corresponding author.
